# Clinical Value of NGS Genomic Studies for Clinical Management of Pediatric and Young Adult Bone Sarcomas

**DOI:** 10.3390/cancers13215436

**Published:** 2021-10-29

**Authors:** Miriam Gutiérrez-Jimeno, Piedad Alba-Pavón, Itziar Astigarraga, Teresa Imízcoz, Elena Panizo-Morgado, Susana García-Obregón, Ana Catalán-Lambán, Mikel San-Julián, José M. Lamo-Espinosa, Aizpea Echebarria-Barona, Marta Zalacain, Marta M. Alonso, Ana Patiño-García

**Affiliations:** 1 Department of Pediatrics, University Clinic of Navarra, 31008 Pamplona, Spain; mgutierrezj@unav.es (M.G.-J.); elenapanizo@unav.es (E.P.-M.); anacatalan@unav.es (A.C.-L.); mzalacaind@unav.es (M.Z.); mmalonso@unav.es (M.M.A.); 2 Department of Pediatrics, Pediatric Oncology Group, Biocruces Bizkaia Health Research Institute, Hospital Universitario Cruces, 48940 Barakaldo, Spain; piedad.albapavon@osakidetza.eus (P.A.-P.); mariaiciar.astigarragaaguirre@osakidetza.eus (I.A.); susana.garciaobregon@osakidetza.eus (S.G.-O.); aizpeabeatriz.echebarriabarona@osakidetza.eus (A.E.-B.); 3 Department of Pediatrics, Faculty of Medicine and Nursing, Campus de Leioa, University of the Basque Country, UPV/EHU, 48940 Barakaldo, Spain; 4 CIMA LAB Diagnostics, University of Navarra, 31008 Pamplona, Spain; timizcoz@unav.es; 5 Department of Traumatology and Orthopedic Surgery, University Clinic of Navarra, 31008 Pamplona, Spain; msjulian@unav.es (M.S.-J.); jlamodeespi@unav.es (J.M.L.-E.); 6 Solid Tumor Program, CIMA, Center for Applied Medical Research and IdiSNA, 31008 Pamplona, Spain

**Keywords:** pediatric sarcomas, personalized medicine, targeted therapy, genomics, NGS

## Abstract

**Simple Summary:**

Clinical management of sarcomas is complex because they are rare and heterogeneous tumors. Management requires a coordinated multidisciplinary approach, especially in children. Genomic characterization of this complex group of tumors contributes to the identification of prognostic biomarkers and to the continued expansion of therapeutic options. In this article, we present the positive experience of two Spanish hospitals in the use of genomic analysis in the overall clinical management of sarcomas in children and young adults. We describe on a case-by-case basis how genomic analysis has contributed to both diagnosis and treatment.

**Abstract:**

Genomic techniques enable diagnosis and management of children and young adults with sarcomas by identifying high-risk patients and those who may benefit from targeted therapy or participation in clinical trials. Objective: to analyze the performance of an NGS gene panel for the clinical management of pediatric sarcoma patients. We studied 53 pediatric and young adult patients diagnosed with sarcoma, from two Spanish centers. Genomic data were obtained using the Oncomine Childhood Cancer Research Assay, and categorized according to their diagnostic, predictive, or prognostic value. In 44 (83%) of the 53 patients, at least one genetic alteration was identified. In 80% of these patients, the diagnosis was obtained (*n* = 11) or changed (*n* = 9), and thus genomic data affected therapy. The most frequent initial misdiagnosis was Ewing’s sarcoma, instead of myxoid liposarcoma (*FUS-DDDIT3*), rhabdoid soft tissue tumor (*SMARCB1*), or angiomatoid fibrous histiocytoma (*EWSR1-CREB1*). In our series, two patients had a genetic alteration with an FDA-approved targeted therapy, and 30% had at least one potentially actionable alteration. NGS-based genomic studies are useful and feasible in diagnosis and clinical management of pediatric sarcomas. Genomic characterization of these rare and heterogeneous tumors also helps in the search for prognostic biomarkers and therapeutic opportunities.

## 1. Introduction

Cancer is one of the main causes of morbidity and mortality in children and adolescents worldwide. The most common cancers are hematological malignancies (leukemia and lymphoma), CNS tumors, and sarcomas (tumors of soft tissue and bone). Sarcomas are rare and heterogeneous, representing around 11.5% of all neoplasms in children and adolescents. The most frequent malignant tumors of bone are osteosarcoma (50%) and Ewing’s sarcoma (40%) [1,2].

The survival of children and adolescents with sarcomas, particularly patients with metastatic disease, has barely improved in recent decades, despite intensification of treatments such as chemotherapy, surgery, and/or radiotherapy. There are more than 100 subtypes of sarcomas, and therefore, differential diagnosis is challenging in some cases. The initial diagnostic process of this group of tumors mostly relies upon immunohistochemistry (IHC) and anatomic location, but many types of sarcomas share similar morphologic features, and their IHC profiles overlap [3]. Several subtypes of sarcomas, however, present specific pathognomonic genetic alterations, such as gene fusions. Unfortunately, detection of these alterations is not possible with the typical techniques and equipment of a pathology laboratory, and requires precise diagnostic techniques at the molecular level [4]. Implementation of new molecular diagnostic techniques makes it possible to obtain a correct diagnosis that enables prior identification of high-risk patients and improved management in certain complex cases, and can be regarded as a further step toward more-personalized medicine in pediatric sarcomas. To be clinically useful, the new techniques need to be fast and work with just a small sample of material obtainable by conventional methods.

Numerous personalized medicine initiatives are already up and running for adult carcinomas (NCI_MATCH trial, IMPACT, LungMAP, and Alchemist), and there is promising development of similar initiatives for the management of pediatric patients diagnosed with hematological cancers [5]. Implementation of the same types of molecular techniques for use with pediatric sarcomas is not, however, so advanced: some types of sarcomas have specific characteristics whose detection requires new and more precise molecular diagnostic tools. Nevertheless, the utility of next-generation sequencing (NGS) techniques in the processes of diagnosis, prognosis, and treatment of sarcomas has been studied and demonstrated [6,7]. In a recent review, Montella et al. [8] summarized the opportunities for biomarker-tailored therapies for most sarcoma subtypes. Grounder et al. conducted a study of 5635 adult and pediatric patients with bone and soft tissue sarcomas, and found that more than half had actionable mutations; 30% of these patients were consequently enrolled in clinical trials [9].

In a multicenter study involving 384 sarcomas, the initial diagnosis was modified in 53 (13.8%) of the patients, leading to a change in the initial management and prognosis of 45 (11.7%) of these patients [10]. In a group of 584 patients with different soft tissue tumors from the AACR Project Genomics Evidence Neoplasia Information Exchange (GENIE) database, a genetic alteration with the potential to influence therapy was identified in 41% of cases [11].

Even more challenging is the management of sarcoma patients without “classical” genetic biomarkers. In those cases, NGS can be used to characterize other molecular “phenotypes” such as tumor mutational burden (TMB), microsatellite instability (MSI), and DNA damage repair scores; for patient stratification, however, the value of such, with even more controversial biomarkers, is controversial in patient stratification [12].

In order to improve treatment options and clinical management for pediatric patients with high-risk and metastatic sarcomas, identification of molecular targets is a priority. NGS tools are outstanding in this role and can identify new biomarkers, find potential molecular targets for new therapeutic drugs, and play a key role in clinical trials [13]. In addition, NGS panels can provide a definitive diagnosis of some heterogeneous sarcomas that are challenging to diagnose by usual pathological approaches.

NGS analysis of sarcomas has detected many somatic alterations that have predictive or prognostic value. In addition, germinal mutations, which are potentially hereditary, have also been found. Thus, NGS already enables improved follow-up management of patients and provides information useful in genetic counseling to the patient and relatives [14].

We examined genomic data from 55 sarcoma samples from 53 pediatric and young-adult patients. Samples were analyzed using the Oncomine™ Childhood Cancer Research Assay (Thermo Fisher, Waltham, MA, USA), a gene panel that identifies point mutations, indels, amplifications, copy number variants (CNVs), and gene rearrangements in 200 genes that are considered cancer drivers. Because Thermo Fisher pipeline analysis does not detect gene deletions, the presence of such alterations was manually determined by two independent observers. The aim of this study was to assess the clinical utility of NGS-based genomic panels in the processes of diagnosis, treatment, and prognosis in pediatric sarcomas.

## 2. Materials and Methods

### 2.1. Patients and Samples

We studied a series of 53 nonconsecutive pediatric and young-adult patients with different sarcoma histotypes: osteosarcoma (*n* = 25), Ewing’s sarcoma (*n* = 16), and other miscellaneous sarcomas (*n* = 12). Pediatric cases were those in which disease presented before the patient was 18 years old. “Young-adult” cases were those in which disease presented between 18 and 31 years of age. All patients had either undergone surgical resection or biopsy at either the Hospital Universitario de Cruces or at the Clínica Universidad de Navarra between 2013 and January 2021. The clinical characteristics of the patients are shown in Table 1. All sarcomas included in the study were histopathologically evaluated on hematoxylin slides, and FISH and IHC techniques, used as primary detection approaches for possible fusion events, were carried out during routine diagnostic procedures with validated reagents according to standard laboratory guidelines.

### 2.2. Extraction of DNA/RNA from Tissues

DNA and RNA was extracted from formalin-fixed paraffin-embedded (FFPE) fresh or frozen tissue using a Maxwell^®^ RSC DNA FFPE kit (Promega, AS1450, Madison, WI, USA) for DNA and a Maxwell^®^ RSC RNA FFPE kit (Promega, AS1440) for RNA. The amount of DNA and RNA was quantified using a Qubit fluorometer, and the amount used for sequencing was 50 ng of either DNA or RNA.

### 2.3. NGS Library Preparation and Sequencing

Tumor profiling to detect sequence alterations and abnormal gene fusions was undertaken using the Oncomine™ Childhood Cancer Research Assay (Thermo Fisher, A36486) according to the manufacturer’s protocol. This tool analyzes the mutational state of 200 genes, including 82 mutation hotspots, 24 CNV targets, 44 genes with full exome coverage (specifically tumor suppressor genes), and an RNA panel for 97 genes (with >1700 fusion isoform variants).

DNA and RNA libraries were generated using Ion AmpliSeq Library Preparation on the Ion Chef System (Thermo Fisher). Complementary DNA (cDNA) synthesis prior to library preparation for the RNA panel was carried out using SuperScript™ VILO™ Reverse Transcriptase (Thermo Fisher). Sequencing was performed using the 540 chips on the Ion Torrent S5 (Thermo Fisher).

### 2.4. Data Analysis

Variants were identified and annotated with the Thermo Fisher Ion Reporter (https://ionreporter.thermofisher.com/ir/) and Oncomine Knowledge Reporter Database (https://www.thermofisher.com/order/catalog/product/A34298).

A filter was included in the bioinformatics analysis to ensure the quality of the generated data (Q > 30). Throughout the analysis process, there were checkpoints for uniformity of the number of reads between samples, alignment percentages, and control of PCR duplicates. This analysis confirmed that the data had the appropriate homogeneity, depth, and consistency for use in a clinical context.

A variant was accepted for analysis if its coverage was at least 500 reads, the variant allele frequency (VAF) was greater than 0.05, and the minor allele frequency (MAF) was less than 0.01. A CNV variant was recognized as such if the amplification was at least ×4 at the 5% confidence level. Fusion genes were recognized as such if they had at least 50 reads.

The identified variants were compared with different databases to analyze the clinical significance of the sequence differences found with respect to a reference genome (hg19). Different web tools and databases were used: Varsome [15], ClinVar [16], OncoKB [17], COSMIC [18], PeCan [19], TumorFusions [20], and PanDrugs [21]. Variants considered to be pathogenic or likely to be pathogenic were visually inspected by using the Integrated Genome Viewer (IGV, https://software.broadinstitute.org/software/igv/) software.

Mutations were represented using the OncoPrinter [22] and MutationMapper [23] tools. Fusions were represented in circos plots generated with the BioCircos package implemented in R.

## 3. Results

### 3.1. Patient and Sample Characteristics

Our cohort consisted of 53 patients diagnosed with sarcoma. Of these, 48 (90.6%) were pediatric patients (under 18 years of age) and 5 (9.4%) were young adults (between 18 and 31 years of age). The median age at diagnosis was 11.8 years. There were 30 males (56.6%) and 23 females (43.4%).

The clinical characteristics of the 53 patients are collated in Table 1. Of the 53 patients, 10 were metastatic at diagnosis. Primary sites included lower extremities (*n* = 22, 41%), upper extremities (*n* = 11, 20.8%), and trunk (*n* = 16, 30.2%). The most frequent sarcomas were osteosarcoma (47.2%) and Ewing’s sarcoma (30.2%); histological types and subtypes are shown in Table 1. Histology was based on 43 samples from the primary site, 10 from metastatic lesions, and 2 from local recurrences.

**Table 1:** Clinical characteristics of 53 patients with sarcoma.

Characteristic	Number (%)
Median age at diagnosis (range)	11.8 (0–30.8)
**Gender**	
Male	30 (56.6)
Female	23 (43.4)
**Ethnic origin**	
European	47 (88.6)
Latin	3 (5.7)
African	3 (5.7)
**Classification of the sarcoma**	
Osteosarcoma	25 (47.2)
Ewing´s sarcoma	16 (30.2)
Other	12 (22.6)
**Pathological subtype**	
Osteosarcoma; *n* = 25	
Osteoblastic	12 (48)
Unknown	5 (20)
Chondroblastic	4 (16)
Osteo-chondroblastic	3 (12)
Parostal	1 (4)
Ewing’s sarcoma; *n* = 16	
Bone	10 (62.5)
Askin tumor	3 (18.75)
Extraskeletal	2 (12.5)
Ewing-like	1 (6.25)
Other sarcomas; *n* = 12	
Undifferentiated pleomorphic sarcoma	2 (16.7)
Chondrosarcoma	1 (8.3, each)
Infantile fibrosarcoma	
Angiomatoid fibrous histiocytoma	
Neurofibrosarcoma	
Synovial sarcoma	
Epitheloid sarcoma	
Desmoplastic small round cell tumor	
Solitary fibrous tumor	
Rhabdoid soft tissue tumor	
Myxoid liposarcoma	
**Location of the primary tumor **	
Lower extremity	22 (41)
Upper extremity	11 (20.8)
Trunk	16 (30.2)
Other	4 (7.5)
**Disease stage at diagnosis**	
Localized	43 (81.1)
Metastatic	10 (19.9)
**Treatment regimen**	
Neoadjuvant chemotherapy	43 (81.1)
Surgery	49 (92.5)
Adjuvant chemotherapy	47 (88.7)
Radiotherapy	29 (54.7)
Targeted therapy	6 (11.3)
Immunotherapy	6 (11.3)
Stem cell transplant	4 (7.5)
**Current status**	
Alive	34 (62.1)
Dead	16 (30.2)
Unknown	3 (5.7)

### 3.2. Genetic Alterations Detected in Sarcomas

**Figure 1:**
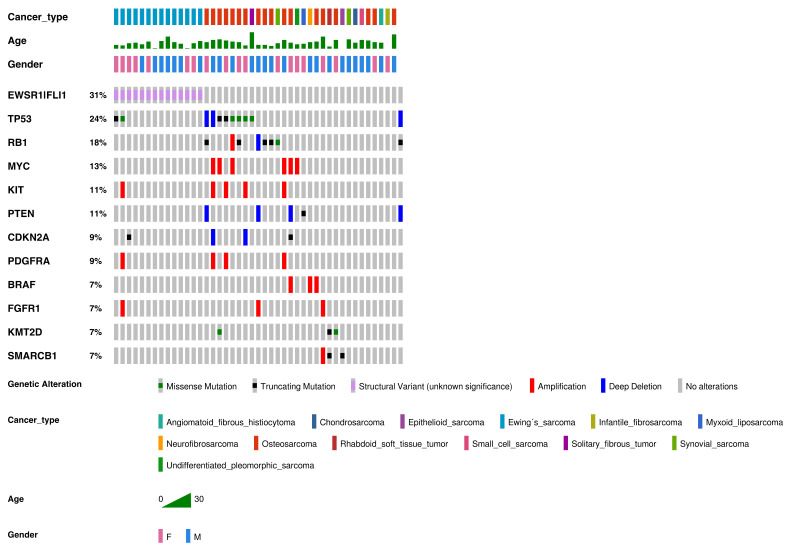
Genetic alterations detected in our cohort of sarcomas (in order of frequency). Genetic alterations, SNVs, indels, and fusions with a frequency ≥7% or three samples. Altered genes identified in two or fewer sarcoma samples were: *ABL2, ACVR1, ARID1A, ARID1B, ASXL1, ATRX, CCND1, CDK4, CDK6, CHD7, CIC, DICER1, ERBB3, FGFR3, GLI1, JAK1, JAK2, IDH1, IGF1R, KDM6A, MET, MYCN, NF1, NF2, NOTCH1, PHF6, PIK3CA, SUZ12, SMARCA4, TCF3, TSC2, WT1,* and rearrangements involving *AGK-BRAF, ARID1B-MIR-4466, BCOR-CCNB3, EGFR-EGFR, ETV6-NTRK3, EWSR1-CREB1, FGFR2-KIAA1217, FUS-DDIT3, HMGA2-LPP, KMT2C-PRKAG2, NAB2-STAT6, RUNX1-MRPS6, SFPQ-EPS15, SS18-SSX2, SS18-SSX4, TMEM178B-BRAF, ZFP36-FOSB,* and *ZC3HAV1-BRAF*.

We analyzed a total of 55 samples from the 53 patients in the cohort; the two additional samples were from metastatic tissue. The most frequently observed genomic alterations are summarized in Figure 1. In total, we detected 151 genetic alterations. The median number of alterations per patient was 2 (range was 0–9), and at least one genetic alteration was identified in 44 of the 53 patients (83%). Of the 151 alterations, 123 were pathogenic, 9 were likely pathogenic, and 19 were variants of unknown clinical significance (VOUS).

Frequent alterations were CNVs (*n* = 52, 34.4% of all alterations), fusions (*n* = 47, 31.1%), missense mutations (*n* = 31, 20.5%), and truncating mutations (*n* = 21, 13.9%). The most commonly altered genes were *EWSR1-FLI1* (31% of all alterations), *TP53* (24%), *RB1* (18%), *MYC* (13%), *KIT* (11%), and *PTEN* (11%).

**Figure 2:**
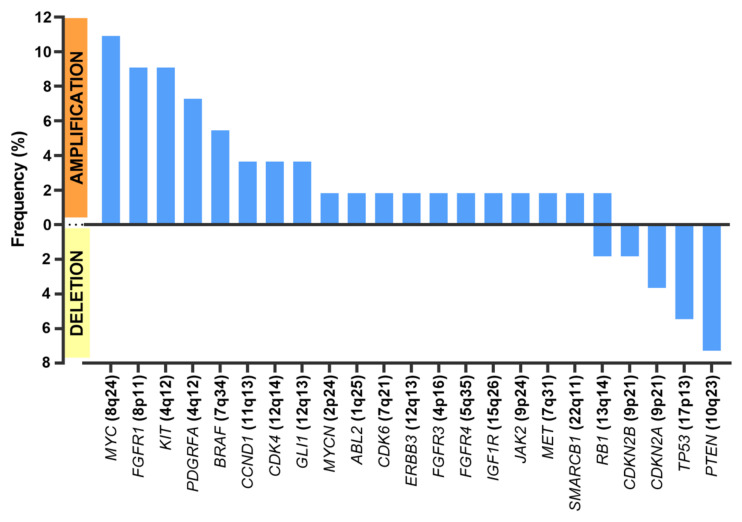
Frequent copy number alterations (CNAs).

Regarding CNVs (Figure 2), *MYC* amplification was present in seven patients (*n* = 7/55 samples (12.7%)), *KIT* amplification was present in five patients (*n* = 5/55 samples (9.1%)), and *PDFGRA* amplification was present in four patients (*n* = 4/55 samples (7.3%)). Because *KIT* and *PDGFRA* genes are neighbors (located at 4q12), amplification was concomitant in all cases but one.

The most frequent deletions affected *PTEN* (*n* = 4/55 samples (7.3% of all samples)), *TP53* (*n* = 3/55 samples (5.5%)), and those encompassing *CDKN2A* and *CDKN2B* (*n*= 3/55 samples (5.5%)).

In most cases, the relationship between CNV and mRNA expression of the genes concerned could not be assessed because the degree of amplification was not high enough, because samples were not fully microdissected and contained normal cells, or because samples were from FFPE tissues and thus extremely scarce. Nevertheless, in the few cases in which assessment was possible—an amplification of *CCND1* in two cases and loss of *PTEN* in another—the CNV finding was validated by a corresponding change in mRNA level as measured by real-time PCR.

**Figure 3:**
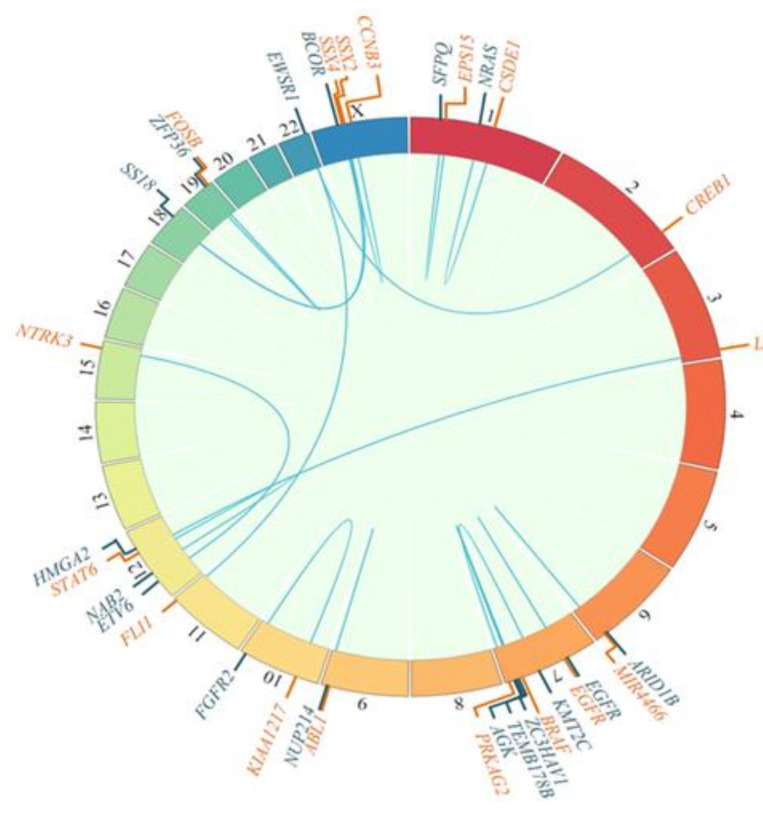
Fusion genes detected, as depicted in circos plots showing 5′ (blue) and 3′ (orange) fusion genes.

Fusions were mostly pathognomonic genetic alterations that provided diagnostic information, as represented in Figure 3.

#### 3.2.1. Osteosarcoma

Osteosarcoma, which affected 25 patients in the cohort, was the most frequent sarcoma in our study (Figure 1; Table 1). The mean age at presentation was 12.3 years (range 5.1 to 26.9 years).

**Figure 4:**
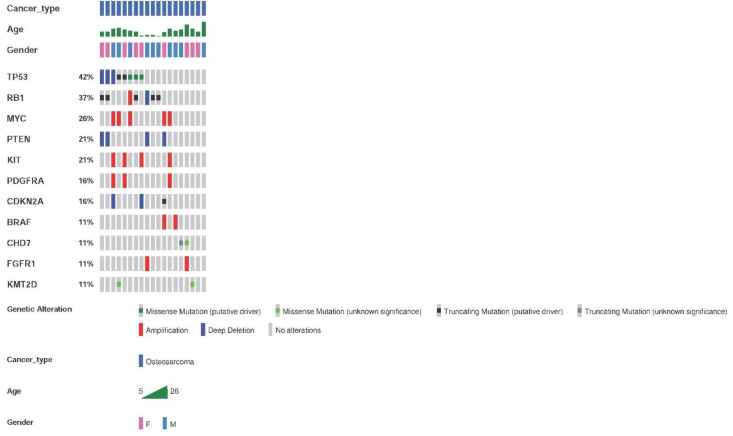
Most frequent genetic alterations in osteosarcoma patients. Genetic alterations, SNVs, indels, and fusions with a frequency ≥11% or two samples. Altered genes identified in less than two sarcoma samples were: *ERBB3, GLI1, CDK4, NOTCH1, KDM6A, ASXL1, SMARCA4, DICER1, SMARCB1, CDK6*, *IGF1R, NF2, TCF3, CDKN2B, ABL2, ARID1B, ARID1A,* and rearrangements involving *FGFR-KIAA1217, HMGA-LPP, KMT2C-PRKAG2,* and *SFPQ-EPS15*.

The genetic alterations detected by the Oncomine^TM^ Childhood Cancer Research Assay (Thermo Fisher) in the 26 samples from 25 patients with osteosarcoma are shown in Figure 4. The most frequent altered genes were *TP53* and *RB1*. Genetic alterations were found in 73.1% of the samples (19/26), with an average of two alterations per tumor.

Regarding CNVs, the most frequently found amplifications contained *MYC*, *KIT,* and *PDFGRA*. Deletions affecting *PTEN* were the second most frequent CNVs. Deletions of *CDKN2A* and *CDKN2B* were also frequent.

#### 3.2.2. Ewing’s Sarcoma

Ewing’s sarcoma, which affected 16 patients in our cohort, was the second most frequent sarcoma in our study (Figure 1; Table 1). The average age at presentation was 11.5 years (range 1.5 to 22.9 years).

The pathognomonic fusion of *EWSR1-FLI1* was found in 14/16 samples (87.5%). Other pathogenic mutations were infrequent. Alterations of *TP53* (three mutations in two samples (18.75% of samples)) and *CCND1* (in two samples (12.5%)) were detected.

#### 3.2.3. Other Rare Sarcomas

The sarcomas in this group were heterogeneous and rarer than osteosarcoma and Ewing’s sarcoma (Table 1).

Genetic alterations were found in 91% of the samples. Known and/or expected genetic alterations that are pathognomonic for the different diseases were frequently identified: *FUS-DDIT3* (myxoid liposarcoma), *ETV6-NTRK3* (infantile fibrosarcoma), *SS18-SSX2* and *SS18-SSX4* (synovial sarcoma), *SMARCB1* mutation (soft tissue rhabdoid tumor), *EWSR1-CREB1* (angiomatoid fibrous histiocytoma), *BCOR-CCNB3* (small cell sarcoma), and *NAB2-STAT6* (solitary fibrous tumor) (Figure 3).

### 3.3. Clinical Relevance of the Genomic Alterations Detected in Pediatric Sarcomas and Clinical Actions Based on the Results of the Genomic Analysis

The genomic analysis led to a definitive first diagnosis in 11 patients, and, in 9 other cases, genomic analysis resulted in a change in the initial cytopathology-based diagnosis. Genomic analysis resulted in a change in therapy in 80% of these 20 cases.

The cases in which there was a change in diagnosis and clinical management are described in this section:

- Case #2. Patient initially diagnosed with Ewing-like sarcoma (negative for *EWS-FLI1* and *EWS-ERG* by FISH analysis). The gene fusion *FUS-DDIT3*, which is the diagnostic hallmark of myxoid liposarcoma, was detected in the genomic analysis. The standard treatment for Ewing’s sarcoma (polychemotherapy, radiotherapy, and autologous hematopoietic progenitor transplant) would have been much more aggressive and potentially toxic than that for myxoid liposarcoma (surgery and local radiotherapy).
- Case #10. Patient initially diagnosed with Ewing’s sarcoma, but was negative for *EWS-FLI1* and *EWS-ERG* by FISH analysis. The genomic analysis confirmed the negative FISH results. The final diagnosis was undifferentiated pleomorphic sarcoma. The most common treatment regimen in Ewing’s sarcoma is polychemotherapy; while in pleomorphic sarcoma, the role of chemotherapy is unclear, and surgical resection is central.
- Case #11. Initial diagnosis was inconclusive: suspected undifferentiated pleomorphic sarcoma or liposarcoma. The definitive diagnosis was solitary fibrous tumor (SFT), identified by the pathognomonic gene fusion: *NAB2-STAT6*. SFT is a neoplasm of intermediate biological potential, and surgical resection alone is the standard treatment in most cases, although in some cases radio or even chemotherapy is indicated.
- Cases #13 and #21. Initial diagnosis was osteosarcoma. The gene fusion *EWS-FLI1,* which is pathognomonic of Ewing´s sarcoma, was detected by genomic analyses. Ewing’s sarcoma and osteosarcoma treatment regimens are different, and treatment for Ewing’s sarcoma frequently involves radiotherapy.
- Case #17. Initially diagnosed with a low-grade tumor after ruling out synovial sarcoma (there was not *SS18* rearrangement). Genomic analysis detected the gene fusion *BCOR-CCNB3*, which, in the new WHO classification, is pathognomonic for BCOR sarcoma, a high-grade tumor. Treatment for BCOR sarcoma includes chemotherapy; surgical resection alone is insufficient.
- Case #40. This patient was positive for SS18 by immunochemistry in the pathological study and consequently diagnosed as having synovial sarcoma. In the genomic analysis, however, *SS18* rearrangement was not detected, and the sarcoma was reclassified as pleomorphic undifferentiated sarcoma. Patients with synovial sarcoma rearrangements might benefit from first-line combination treatment of ifosfamide with doxorubicin.
- Case #49. Initial diagnosis was Ewing´s sarcoma. There were no *EWSR1* rearrangements in the genomic panel, but two truncating mutations were present in *SMARCB1*. The diagnosis was changed to rhabdoid soft tissue tumor, which requires intensive multiagent chemotherapy, and could benefit from a selective EZH2 inhibitor.
- Case #50. Initial diagnosis was Ewing´s sarcoma, as the patient was positive by FISH for the classical *EWSR1* rearrangements. The patient underwent four cycles of treatment according to the EuroEwing 2012 protocol before genomic analysis detected the *EWSR1-CREB1* rearrangement, the diagnosis was changed to angiomatoid fibrous histiocytoma, and chemotherapy was suspended. Radiotherapy was continued.

The other two cases in which a definitive diagnosis was reached by genomic analysis were two Ewing’s sarcomas in which the genomic analysis detected the pathognomonic rearrangement *EWSR1-FLI1,* while routine studies did not. Thus, NGS detected the *EWSR1-FLI1* rearrangement in 87.5% of the samples (14/16) by NGS, while more conventional pathological techniques detected it only in only 75% (12/16) of cases.

These cases highlight the importance of obtaining genomic results when they are needed clinically, and thus the need for well-coordinated, well-managed multidisciplinary teams.

### 3.4. Detection of Potentially Actionable Genomic Alterations

We used the OncoKB tool developed by the Memorial Sloan Kettering Cancer Center [17] to classify the predictive value of the genomic alterations identified. According to this tool, of our sample of tumors from 53 pediatric and young-adult patients, 2 patients (3.8%) had a genetic alteration with a FDA-approved targeted therapy (OncoKB Level 1), and 16 patients (30%) had at least one potentially actionable alteration (Levels 2 or 3) (Table 2).

Two of our patients had a Level 1 indication for treatment upon the identification of a genetic biomarker. OncoKB Level 1 identifies a FDA-recognized biomarker predictive of response to an FDA-approved drug. One of the patients concerned had a neurofibrosarcoma, in relationship to his underlying disease, neurofibromatosis type 1. *NF1* mutation was detected; selumetinib (a MEK inhibitor) is an approved treatment for patients diagnosed with neurofibromatosis type 1 with neurofibroma-related complications [24,25]. This patient died of disease progression before initiation of treatment with selumetinib. The other patient had a diagnosis of infantile fibrosarcoma, and *ETV6-NTRK3* fusion was detected in the tumor. Patients with NTRK fusion-positive solid tumors have two approved therapeutic options: entrectinib [26,27] and larotrectinib [28,29], which are potent inhibitors of tropomyosin receptor kinase (TRK). This patient initially received conventional treatment, which failed to stop disease progression, but was then included in a phase III clinical trial of targeted therapy with larotrectinib and achieved complete response.

The 16 patients with Level 2 or 3 alterations were treated according to standard protocols rather than with targeted, biomarker-driven treatment.

Analysis of these cases is interesting in the search for predictive biomarkers of response to existing drugs used in another indication, as well as for new targeted drugs based on robust biological data. In addition, recurrent sarcomas harboring genomic alterations (mainly Levels 1–3) open up therapeutic possibilities in patients with poor prognosis under conventional treatment.

## 4. Discussion

The 5th WHO classification of bone and soft tissue sarcomas, published in 2020, stresses the importance of the use of genomic tools (NGS-based panels), particularly those that facilitate differential diagnosis, for the management of sarcomas [30]. These panels make it possible to improve the classification of sarcomas previously grouped into families, such as Ewing-like tumors; they can also detect previously unreported genetic alterations, which have potential diagnostic, prognostic, and therapeutic value [4].

Our study highlighted the need to implement these panels in routine clinical practice, since, in 18.6% of our patients, the genomic panel made it possible to reach a correct initial diagnosis (where conventional pathological methods had failed), or indicated that it was necessary to change an existing diagnosis, leading to a change in therapy in 80% of those in whom a genetic alteration was detected or ruled out. In addition, three of our patients benefited from targeted therapies based on genomic findings, and a further four patients could have received targeted therapy if the drug had been available at the time of diagnosis.

The GENSARC study had a similar outcome to ours; NGS data affected the definitive diagnosis in 14% of cases for which a histological diagnosis was made by a pathologist specialized in sarcomas [10].

Sarcomas are rare and heterogeneous tumors for which diagnosis based on a single small Tru-Cut biopsy can be a challenge. For these reasons, although conventional pathology continues to be the mainstay in their diagnosis, there is unquestionable diagnostic value in the use of NGS-based genomic studies. Genetic analyses are even more useful if there is disease progression, either local relapse or distant metastasis, when detailed genetic analysis of a patient’s tumor can be of signal importance by determining whether there is an appropriate targeted therapy. This is relevant, since almost half of the patients relapse and eventually die, with the treatment for disease-progression having palliative rather than curative purposes in most cases, and genetic analysis might identify biomarkers that could have an impact on the management of patients during treatment and in the event of disease progression [31,32].

Up to 30% of the genetic events in our sample are potentially actionable according to the OncoKB precision oncology knowledge base. In addition, in 83% of the cases, the genetic alterations were of potential value either in providing insight into the biological behavior of the tumor, or as prognostic markers or therapeutic targets. For example, in patients with *NTRK* fusion-positive sarcomas, the response to selective inhibitors entrectinib [26,27] and larotrectinib is usually so striking that, for tumors such as infantile fibrosarcoma, some authors propose their use as first-line treatment [28,29]. In this study, we observed a complete response to larotrectinib in a refractory and aggressive neonatal fibrosarcoma.

Several studies (Peds-MiOncoSeq, iCAT, BASIC 3) [33,34,35,36,37] have reported percentages of *potentially actionable* findings in the range of 21–51% in samples analyzed from pediatric and adult patients diagnosed with different solid tumors, including sarcomas. There is ambiguity in the definition of a potentially actionable genetic event; in our study, only drug-treatable genomic alterations were considered actionable, and consequently the percentage might be underestimated. Given the ambiguity and the continuous developments in this field, genomic data should be regularly reviewed.

There are myriad reasons why, in practice, pediatric patients cannot access bi-omarker-driven therapies. In this series, given the retrospective design, many patients did not have a treatment/trial or even NGS genetic testing during the period of clinical utility. Other reasons include nonavailability of a pediatric clinical trial for the relevant molecu-lar target (although the number of trials is steadily increasing), cost, family reasons, and nonfulfillment of clinical criteria for trial inclusion. We recently reviewed early-phase pediatric oncology trials in Spain from 2007 to 2020, and concluded that, even with improvements driven by a new legal framework, involvement in international research networks, and multidisciplinary cooperation (between academia, regulators, patient advocacy groups, and pharmaceutical industry), it is still necessary to address barriers for children of all ages and diagnoses to access new drugs [38].

In the new WHO classification [30], there is no Ewing’s sarcoma chapter as in previous versions. This reflects a more accurate biological understanding of the complex genomic landscape of this updated category. On the basis of genomic alterations, a distinction is now made between four types of undifferentiated small round cell sarcomas (USRCS) of bone and soft tissue: Ewing’s sarcoma (*EWSR1-FLI1*, *EWSR1-ERG,* or *EWRS1-ETS),* round cell sarcoma with *EWSR1*-non-ETS fusions (*EWSR1-NFATCD2* or *EWSR1-PATZ1*), sarcomas with positive rearrangement of *CIC* (*CIC-DUX4*), and sarcomas with genetic alterations in *BCOR* (*BCOR-CCNB33*). There remains a small subset of round cell sarcomas that cannot yet be classified and will continue to be referred to as Ewing-like. USRCSs can only be diagnosed by using a genomic panel that includes all possible rearrangements. The necessity of a genomic panel for USRCSs opens up the possibility of extending the panel for diagnosis of other entities [39].

In the latest WHO classification of sarcomas, it is not only the Ewing’s sarcomas that have been reclassified on the basis of genomic characteristics, and new types of epithelioid hemangioendothelioma are described based on rearrangements (*WWTR-CAMTA1* and *YAP1-TFE3*). New entities, such as the fibroblastic tumor (with a *EWSR1-SMAD3* rearrangement), have also been established. Other types, such as round cell liposarcoma, are no longer recognized. In our study, in addition to the classical rearrangements, we detected the fusion *BCOR-CCNB3*, which defines a tumor subtype; the pathognomonic fusion *NAB2-STAT6*, which is more relevant in the latest WHO classification than previous versions; and the *ETV6-NTRK3* fusion, which has approved drug treatments.

In bone sarcomas that are metastatic, refractory to treatment, or early-recurrent, the prognosis is dismal. To date, there is no curative treatment available for most patients with metastatic sarcomas, and there is no agreed-upon standard approach for chemotherapy treatment. Prognostic molecular biomarkers that define this high-risk patient population are urgently needed. Research is also pressingly required to elucidate the molecular pathways that are involved in pathogenesis and to which new treatments might be targeted [40]. To meet these research goals, it is necessary to add genomic analysis based on NGS to the usual diagnostic techniques, FISH, or RT-PCR. NGS-based genomic analysis is also needed because most pathological anatomy laboratories do not test for rare rearrangements, and therefore a negative FISH or RT-PCR does not rule out a diagnosis of Ewing’s sarcoma.

Ewing’s sarcoma diagnosis is a big challenge for those cases that are negative for all rearrangements. A related challenge is recognizing the exact nature of a rearrangement. For example, in our study, a tumor with a rearranged *EWSR1* detected by FISH was initially diagnosed as Ewing’s sarcoma until genomic analysis revealed a *EWSR1-CREB1* rearrangement, and the diagnosis was consequently changed to angiomatoid fibrous histiocytoma. This kind of difficulty stems from the substantial overlap in the clinical and morphological features of different tumor types, together with the low incidence rate. In addition to availability of a complete set of diagnostic tools, diagnosis demands a multidisciplinary team [41]. The consequences of misdiagnosis can be considerable. For example, in our study there was a tumor initially thought to be osteosarcoma, but it turned out to be Ewing’s sarcoma (NGS genomic analysis detected an *EWSR1-FLI1* rearrangement); this directly affected management of the patient’s treatment. In short, genomic analysis is recommended to obtain the diagnosis and to select and adjust the therapeutic scheme, but is required if PCR or FISH do not detect an expected diagnostic rearrangement. In addition, genomic analysis can identify somatic mutations relevant for the understanding of tumor development, progression, and prognosis.

Interpretation of the results of our study should take into account the partially retrospective design, the small sample size, and the wide variety of sarcoma subtypes included. Nevertheless, the paucity of cases and heterogeneity of sarcoma subtypes also represents the real clinical setting of most hospitals in many countries, where a centralized pediatric pathological review is difficult to achieve, and thus, we think that our study gains weight in the current-to-date management of pediatric sarcomas and emphasizes the need of availability of genomic studies for all patients.

## 5. Conclusions

NGS-based genomic studies are both feasible and useful for diagnosis and clinical management in children and young adults with sarcomas. In addition, genomic characterization of these rare and heterogeneous tumors contributes to the identification of prognostic biomarkers and to the continued expansion of therapeutic options, either by making it possible to participate in clinical trials of existing targeted drugs or by providing data on which to base the search for new targeted therapies. In both the here and now and in the future, NGS-based genomic analysis promises to improve the prognosis for these young patients.

## Figures and Tables

**Table 2:** Potentially actionable alterations identified by OncoKB in 53 sarcomas.

Gene	Type of Alteration	*N* Cases	OncoKB Level	Drugs
***NF1***	**Truncating mutation**	**1**	**Level 1**	**Selumetinib**
***ETV6-NTRK3***	**Fusion**	**1**	**Level 1**	**Larotrectinib**
***CDK4***	**Amplification**	**2**	**Level 2B**	**Palbociclib, abemaciclib**
*KIT*	Amplification	5	Level 3B	Imatinib, sunitinib, regorafenib, ripetrinib
*PDGFRA*	Amplification	4	Level 3B	Imatinib, sunitinib
*BRAF*	Fusion	3	Level 3B	Cobimetinib, trametinib
*IDH1*	Mutation missense	1	Level 3B	Ivosidenib
*MET*	Amplification	1	Level 3B	Cabozantinib, crizotinib
*FLI1*	Fusion	14	Level 4	TK216
*PTEN*	Deletion/Truncating mutation	5	Level 4	AZD8186, GSK2636771
*CDKN2A*	Deletion/Truncating mutation	4	Level 4	Abemaciclib, ribociclib, palbociclib
*FGFR1/FGFR3*	Amplification	4	Level 4	AZD4547, erdafitinib, BGJ398, Debio1347
*SMARCB1*	Fusion/Truncating mutation	2	Level 4	Tazemetostat

The first 3 bolded rows highlight the only three cases with direct treatment indication according to evidence level <3.

## Data Availability

The data presented in this study can be available on request from the corresponding author. The data are not publicly available because they correspond to pediatric cancer patients as part of their clinical management.
